# Generative AI tools in reflective essays: Moderating moral injuries and epistemic injustices

**DOI:** 10.4102/safp.v67i1.6123

**Published:** 2025-08-29

**Authors:** Nontsikelelo O. Mapukata

**Affiliations:** 1 School of Public Health, Faculty of Health Sciences, University of Cape Town, Cape Town, South Africa

**Keywords:** artificial intelligence, academic literacy skills, epistemic injustice, health sciences students, moral injury, reflective essays

## Abstract

The emergence of large language models such as ChatGPT is already influencing health care delivery, research and training for the next cohort of health care professionals. In a consumer-driven market, their capabilities to generate new forms of knowing and doing for experts and novices present both promises and threats to the livelihood of patients. This article explores burdens imposed by the use of generative artificial intelligence tools in reflective essays submitted by a fifth of first-year health sciences students. In a curriculum centred around Vision 2030 at a South African university, deviations from prescribed guidelines in an essay requiring students to demonstrate an understanding of the models of disability are presented as moral injuries and epistemic injustices. Considering our obligations as educators to contribute to a humanising praxis, the author evaluates an eroded trust between educators and students and offers an interim solution for attaining skills in academic literacy in a developing country.

**Contribution:**

This article provides health sciences educators with an opportunity to pause and reflect on how they would like to integrate generative AI tools into their assessments.

## Introduction

Computational linguistics, in the form of large language models (LLMs) such as ChatGPT and other generative artificial intelligence (AI) tools, contributes significant advances likely to transform the knowledge production and consumption processes in the health care industry.^1,2,3,4^ A significant uptake in e-health is recorded in the post-pandemic phase in health sciences education, performance approaches in research-related activities, and the practice of medicine.^1,2,3,5^ In a 2018 publication, Mahomed cited opportunities presented by AI on the future of medicine, particularly in developing countries, while also cautioning on potential harms including ethical, legal and social challenges.^6^ Concerns expressed by Mahomed resonate with others who express reservations regarding the limitations of LLMs, ethical issues concerning ownership, trustworthiness and privacy, the lack of guidelines, and the absence of a uniform definition of AI.^2,3,7^

Given that health sciences programmes are content-heavy, complex, and thus time-consuming, becoming a health professional in an era of digital technologies must respond to calls for integrating AI into higher education processes.^4,7^ Access to machine learning tools that simulate human intelligence, assist with text generation, text summarisation and text correction.^8^ An assumption is that time gained using AI tools can be better utilised to navigate the complexities of learning and afford students opportunities to keep up with medical advances, ultimately contributing to improved patient outcomes.^1,2,5^ However, this is not without challenges. The volume of results sourced through generative AI tools has not been sufficient to institute quality assurance measures in time to test their validity, reliability and effectiveness in assessments. Thus, this article aims to reflect on burdens imposed by a fifth of first-year health sciences students (FYHSS) registered for professional degrees at the University of Cape Town (UCT), who used generative AI tools in reflective essays.

## Contextualising professionalism in the artificial intelligence era

The core curriculum for FYHSS includes two compulsory semester courses, ‘Being a Professional’ and ‘Becoming a Health Professional’ taken during the first academic year of a 4-year or 6-year degree.^9^ A commitment to socialise health sciences students to future practice is premised on an understanding of the attainment of professionalism not as an event but a process facilitating the transition of students from lay persons, apprentices, and novices on a journey to becoming health care professionals (HCPs) who will become experts in their fields.^10,11^

Firstly, an understanding of moral values as norms and standards expected of health sciences students flows from the modified Hippocratic Oath students are introduced to as part of the first-semester course – Becoming a Health Professional. Secondly, students are introduced to the Standards of Good Practice as part of registration with the Health Professions Council of South Africa (HPCSA). Thirdly, students are allocated to groups of 12 and sign a group contract with designated teaching assistants^i^ (TAs – commonly known as facilitators). This governs their attitudes and behaviours in a shared space, specifies acceptable conduct and outlines terms of reference regarding their learning outcomes.

Although the teaching on professionalism is focused in the first year, it is informed by an understanding that learning about professionalism takes both explicit and implicit forms and is facilitated through practice application by various stakeholders. These include clinical educators, health care workers, community stakeholders, patients, mentors and role models. Beyond graduation, it is facilitated through lifelong learning and intrinsic motivation.^12^ This reinforces an expectation by members of the public to require all HCPs to demonstrate an understanding of contextual attributes of professionalism. As this is a non-negotiable requirement by professional bodies, HCPs are expected to maintain a high moral standard and meet professional standards of practice.^12^

Given these professional obligations, in their first year, core content for FYHSS includes an introduction to the ethics of health care practice, social media and professionalism in the digital age, communication skills, diverse populations, human rights in health, public health, primary health care, environmental health, and disability and impairment. Professionalism is considered a core construct in a curriculum that facilitates interprofessional education and collaborative learning for 450 students. Students must master medical writing early in the pre-clinical phase.^9,13^

**FIGURE 1:**
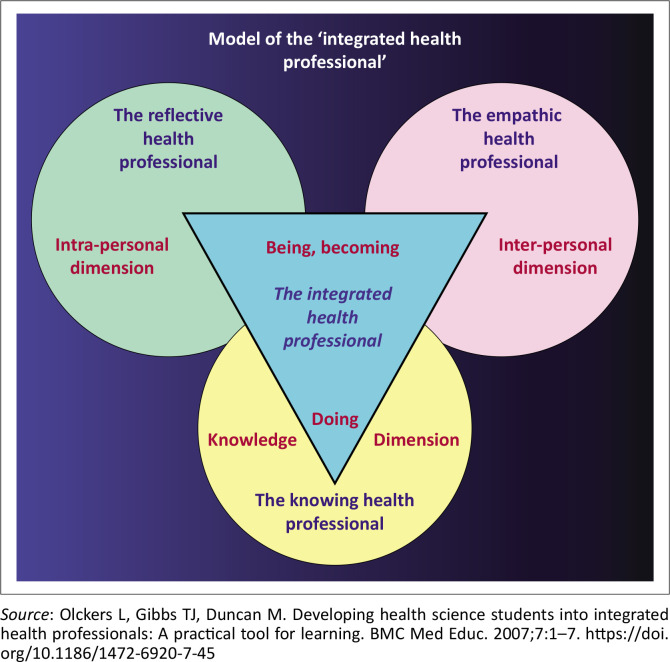
The integrated health professional (IHP) model.

This demands a cascading exposure of novice FYHSS to multilevel assessment approaches to achieve the desirable graduate competencies. In their first semester of year 1, they draw from an integrated health professional (IHP) model (Figure 1) by Olckers et al. as a theoretical framework.^14^

Accordingly, for each topic, an evolved understanding of professionalism in practice requires FYHSS to demonstrate that they are knowledgeable in line with the knowledge dimension. The interpersonal dimension facilitates a demonstration of their empathy, while the intrapersonal dimension facilitates their capability to reflect as emerging health professionals who are morally sensitive and culturally astute.^14^ These competencies are tested in a range of assessments, and the reflection is integrated into their learning experiences in small groups and also through individual activities.^10,14^ First-year health sciences students must also attain basic information, academic and digital literacy skills.

Since 2023, teaching and learning have also included structured sessions on innovative approaches such as machine learning. Responding to the demands of a transforming curriculum framed around Vision 2030, the teaching approach focuses on graduating students who are socially accountable and can manage common presenting problems in their communities while sustaining the University of Cape Town’s (UCT) research profile. Thus, besides introducing students to prescribed content, educators must acknowledge the diversity of their profiles and the multiplicity of contexts in which they undertake their pre-university education.

Given the structural inequities in our schooling system, Fullan and Langworthy implore educators to employ deep learning pedagogies preparing students to become lifelong learners and collaborative problem solvers.^15^ In that regard, beyond the 2015/2016 #FeesMustFall students’ protests, educators were conscientious about adopting a teaching and learning approach structured around decolonised curricula. Drawing from a humanising praxis as a pedagogy of teaching and learning, FYHSS are co-opted through a series of activities to become co-constructors of knowledge, and their learning is centred around a practice approach grounded in real-life experiences.^9^

## The attainment of academic literacy skills

Following on earlier assertions about a humanising praxis, annually, in preparing for the reflective essay on living with disability, the delivery follows a team-teaching approach. Students are introduced to disability as a diversity and equity issue in various sessions, including a didactic lecture and a plenary session focusing on the three models of disability (the medical, the social and the biopsychosocial). This plenary is delivered as a lived experience by a content expert, an academic, and a C4 spinal cord injury survivor who is also a husband and a father. As part of the lecture, he shares aspects of his personal life so students can appreciate what it means to live with a disability.

This is followed by a facilitated 2-h group session, where 12 students from five professional disciplines explore different perspectives on the meaning of disability. By the end of the session, they should demonstrate an understanding of the models of disability. Before submitting the essay for marking, students attended a refresher session that includes a 20-min information literacy workshop offered by colleagues in the library, and a 45-min academic literacy workshop provided by the Writing Lab.

In 2024, during a 12-week teaching cycle for the second-semester course, students had 5 h of structured engagement for an essay assessment, which contributed 25% to their year mark. For this activity, a take-home, open-book assessment, each student submitted a reflective essay with a prescribed word count in response to a detailed case-based scenario. Students have access to guidelines on how to approach a reflective essay and the marking rubric. They had already demonstrated essay writing as a competency as part of the first-semester course, which is a prerequisite for the second-semester course. Applying the humanising pedagogy, in addition to students demonstrating an application of their theoretical knowledge in practice in two of the three models, students were required to reflect on observations of experiences of people living with disabilities in their communities. Recognising the generative capabilities of machine learning and the plethora of AI tools available online (free text humaniser, free generator, AI rewriter, etc.), for this essay, students were cautioned not to use AI tools: ‘Using artificial intelligence tools to write either part/s of or the entire essay is prohibited. No marks will be awarded for dictionary definitions and anonymized authors’.

**FIGURE 2:**
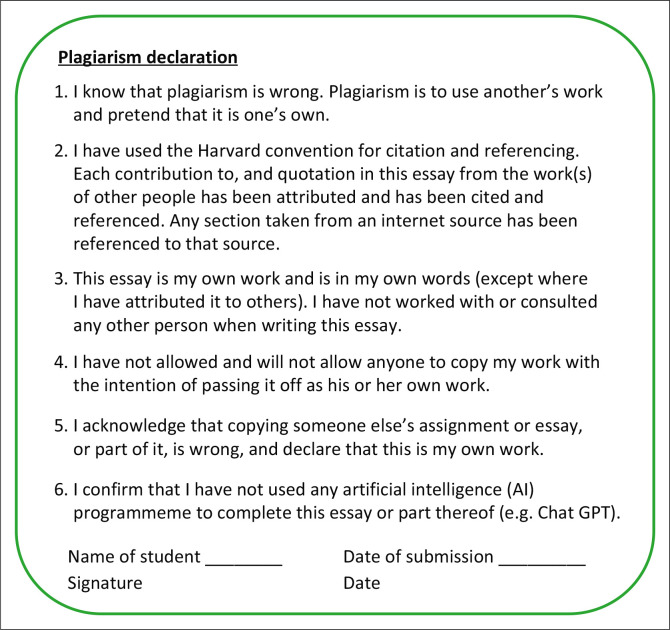
Plagiarism declaration statement.

Students must include an itemised plagiarism declaration statement for each assessment submission, whether an individual or group activity (Figure 2). This serves as a prompt to ensure students comply with the assessment guidelines.

On submission to the designated learning management system (LMS), each essay was subjected to a plagiarism detector, and a Turnitin score was recorded based on a similarity index to previous essays or other work from other institutions.^8^ In a programme structured around small group learning, TAs mark their group’s 12 essays, and the course convenor (the author) moderates all the groups in a structured algorithm, resolves mark disputes and addresses specific requests, such as instances where a student deviates from the brief; in this instance, the use of AI tools.

## Artificial intelligence imposed burdens in reflective essays

A period of four weeks was set aside for marking and moderation of essays. Within the first week of marking, TAs flagged transgressions in some essays. TAs reported encountering essays that appeared to be written by more than one person:

[*O*]ne part of the essay was posh and appeared to be written by a post-graduate student and not a first-year, but the other section appeared to be written by the student. (TA 1)

There would be quite a few of these emails from experienced TAs. A common theme in these emails was the violation of the group contract discussed at the beginning of the semester, where each student contributed to the terms of reference regarding their conduct and understood the role of the TA in supporting the learning experience, as well as the boundaries that must be observed.

On the dedicated LMS, the Turnitin plagiarism similarity index is visible on the landing page. On the other hand, the AI detector is inaccessible and almost hidden from the student’s view. Whether the detection rate was 43%, 35%, or 29%, it came with a disclaimer and a warning suggesting that the text was likely generated from an LLM and should be treated cautiously. Based on the detection rate on the LMS, it was evident that a fifth of the class (*n* = 90) used AI tools despite several written warnings and reminders, which imposed an array of untold ethical dilemmas on the team. These deviations, as detected in a few essays, were unjustifiable. For example, the IHP model developed by Olckers et al.^14^ as a reflection tool specifically for our programme was replaced by the Integrated Health Partnerships (IHP) model, a health system of care developed by the Minnesota government in the United States of America. At times, the generative capacity of AI could only be compared to a truck with no brakes, for example, ‘The second medical model is known as the social model’. Other indicators in essays that relied on generative tools included the use of different font sizes (> 12) in the same essay, failure to use prescribed fonts (students had three to choose from – Arial, Calibri or Times New Roman), and a lack of cohesion, ultimately resulting in their inability to apply theory to the given scenario.

**FIGURE 3:**
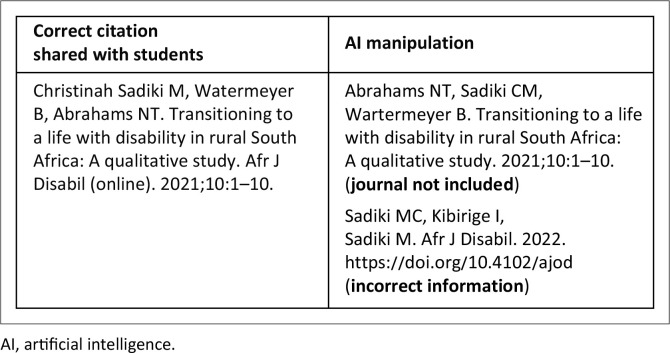
An example of an artificial intelligence-manipulated citation.

In their study guide, FYHSS can access a list of prescribed and recommended readings. Yet, several students relied on AI-generated reference lists that were either incomplete or manipulated to appear different. The references were listed incorrectly or were missing critical information, such as the journal’s details or limited authors’ information (Figure 3).

It was also not uncommon to have in-text references that did not match the reference list, and some students submitted a long list of references they had not cited in their write-up. A few included blogs that did not match their arguments about disability.

Given the above and the ambiguity related to AI-generated text, our School decided not to implement the academic dishonesty policy, which would automatically award a zero and prompt educators to file a complaint with the Student Tribunal. Tasked with advancing the educational development of staff and students, we considered counsel offered by UCT’s Centre for Higher Education Development. They cautioned against punitive approaches as consultations were underway to inform a university policy. Instead, essays that returned an AI score based on content presented or other irregularities were awarded a mark between 50% and 58% depending on the extent of dependency (a detection rate above 50%) and efforts to mask wrongdoing. For example, a few students (> 10) submitted a PDF version of the essay to mask the manipulation instead of the standard Word document. Observing deviations and patterns, and linking them to established groups, was possible because the author had access to the entire class’s essays. This was evident in essays that presented similar content, with the same errors, including missing in-text references. This was a significant finding, particularly in those flawless essays that generated high scores for potential AI use for two-thirds of the essay (50% – 60% AI detection rate).

## A moral injury and an epistemic injustice

The incidents cited in this article reflect a moral injury that extends beyond the individual,^1,2,3,4^ highlighting the potential harms of LLMs.^6^ With the policy in the developmental phase, managing AI-supported essays was labour-intensive. There was a shift in role from an education enterprise crafted with a detailed marking rubric shared with FYHSS to a detective exercise without pedagogical guidelines. Deng et al. reported many limitations cited in this reflection, but did not address the effect on educators.^3^

In this article, employing AI tools contravened course rules and violated the bedrock of social accountability, as FYHSS must integrate their understanding of content into future practice. As primary markers, a few TAs doubted their capabilities as they faced a distorted reality. As some of the TAs and the author had to work through several essays that employed AI tools, the 2-h weekly training had to be extended to include debriefing sessions. De Villiers-Botha argues that planning for higher education content in South Africa and elsewhere is a regulated and complex exercise that should not be compromised by the advent of new technologies.^5^ Although the number of violations was estimated to be a fifth of the class (*n* = 90) based on detection reports on our LMS, the reality may be slightly more, as some students used AI tools to aid the essay writing experience. In contrast, others delegated their responsibility to machine learning, a phenomenon reported by Barron.^8^ This manipulation included substituting the IHP model as a class-based theoretical framework with a US-based IHP model that directs practice-based interventions in Minnesota, US. Notable was the fabrication of information about the models of disability, with a few essays leaning on theological constructs and artistic satire that was sometimes offensive. Kay et al. reported these acts as evidence of epistemic injustices^16^ that are not aligned with the transformation agenda of Vision 2030, as it seeks to ‘unleash human potential in a fair and just society’, while Deng et al. posited these as inherent risks and challenges of LLMs.^3^ Consequently, these acts violated the trust between educators and students, encroaching on educators’ role as content experts. Because of the complexities of grading the essays, they also undermined the university’s efforts to contribute to authentic knowledge acquisition and academic literacy skills. De Villiers-Botha argues that inherent biases linked to using AI tools as substitutes for quantitative measures of human capacity are likely to interfere with indicators of academic success.^5^ In the current format, where students wrote the essay as a take-home assessment, Barron argues that we inadvertently promoted plagiarism by granting students access to AI tools.^8^ In our case, we assumed all students would follow the assessment guidelines. Considering that we do not have valid and reliable instruments to measure the AI literacy skills described by Laupichler et al.,^7^ higher grades may be awarded to students who made no effort to write an essay violating the rights of those who did. Meng et al. believe that the use of AI tools contravenes the goals of academic writing.^2^

The selective punitive approach, in which we awarded some marks to students who used AI tools despite the course rules, considered the status of first-year students as novices. In their first year at university, their moral development is primarily influenced by their upbringing, the school environment, the external environment in the form of social media and the heightened marketing of the values associated with AI tools. The university may not yet have much influence in developing a moral conscience in first-year students in a context where there are only simulated patient interactions, as was the case with the case scenario linked to this essay. In an article published in 2014, the authors outlined the development of personal attributes of health sciences students and expounded on the value of clinical rotations and how these experiences influence the internalisation of the Oaths.^12^

## Way forward

A fair and just approach is one where educators step onto a moral higher ground. Therefore, considering our obligations to equip students with information, digital and academic literacy skills, open-book tests should be limited to low-stakes assessments using the IHP model as our moral compass.^14^ As we continue to explore pedagogical options, students will be required to write their reflective essays in a controlled environment, as trust, respect, accountability and integrity are central constructs in becoming a health professional.^10,11,12^ This mediation is supported by McKenna and Tshuma^17^ who caution academics to be mindful of the students’ vulnerability to AI, their blind loyalty and a false confidence that influences students to abandon their commitment to learning about academic writing. The two authors support our proposed plan to adopt a dual approach that focuses on knowledge generation. Laupichler et al. presented valid and reliable instruments to assess AI literacy skills through comparative self-assessments.^7^ Such measures and others suggested by other scholars can be used to upskill educators in appreciating the value of AI-supported tools. Primarily, in a transforming curriculum, there are opportunities to explore collaborative partnerships with colleagues across faculties as we seek to fulfil the goals of Vision 2030. Lastly, educators must be cognisant of their mandate and ensure that students attain the desirable competencies to serve a population with diverse health needs.

## Conclusion

This article sought to shed light on course-specific AI-induced events, their influence on the learning experiences of some of our students, and their impact on the relationship between students and educators. Through reflectiveness, the author highlighted the obligations of educators in equipping a future cohort of HCPs with the requisite knowledge and skills. The recommendations presented in this article are not prescripts for responding to emerging technologies but mere suggestions for reducing moral injuries and limiting epistemic injustices.

## Footnotes

i Teaching assistants (TAs), commonly known as facilitators, include independent board-certified health care professionals and postgraduate students (Master’s and Doctoral candidates). They are drawn mainly from two faculties: humanities and health sciences and often perform as TAs in more than one programme. Their teaching experience ranges from 12 months to 10 years. As part of our programme, TAs participate in weekly training sessions, including training on how to mark essays.
